# Patient-specific finite element estimated femur strength as a predictor of the risk of hip fracture: the effect of methodological determinants

**DOI:** 10.1007/s00198-016-3597-4

**Published:** 2016-04-23

**Authors:** M. Qasim, G. Farinella, J. Zhang, X. Li, L. Yang, R. Eastell, M. Viceconti

**Affiliations:** 1 Department of Mechanical Engineering, University of Sheffield, The Pam Liversidge Building, Mappin Street, Sheffield, S1 3JD UK; 2 INSIGNEO Institute for in silico Medicine, University of Sheffield, Sheffield, UK; 3 Auckland Bioengineering Institute, University of Auckland, Auckland, New Zealand; 4 Department of Oncology and Metabolism, University of Sheffield, Sheffield, UK

**Keywords:** Bone strength, Finite element analysis, Hip fractures, Osteoporosis

## Abstract

**Summary:**

A finite element modelling pipeline was adopted to predict femur strength in a retrospective cohort of 100 women. The effects of the imaging protocol and the meshing technique on the ability of the femur strength to classify the fracture and the control groups were analysed.

**Introduction:**

The clinical standard to estimate the risk of osteoporotic hip fracture is based on the areal bone mineral density (aBMD). A few retrospective studies have concluded that finite element (FE)-based femoral strength is a better classifier of fracture and control groups than the aBMD, while others could not find significant differences. We investigated the effect of the imaging protocol and of the FE modelling techniques on the discriminatory power of femoral strength.

**Methods:**

A retrospective cohort of 100 post-menopausal women (50 with hip fracture, 50 controls) was examined. Each subject received a dual-energy absorptiometry (DXA) exam and a computed tomography (CT) scan of the proximal femur region. Each case was modelled a number of times, using different modelling pipelines, and the results were compared in terms of accuracy in discriminating the fracture and the control cases. The baseline pipeline involved local anatomical orientation and mesh morphing. Revised pipelines involved global anatomical orientation using a full-femur atlas registration and an optimised meshing algorithm. Minimum physiological (MPhyS) and pathological (MPatS) strengths were estimated for each subject. Area under the receiver operating characteristic (ROC) curve (AUC) was calculated to compare the ability of MPhyS, MPatS and aBMD to classify the control and the cases.

**Results:**

Differences in the modelling protocol were found to considerably affect the accuracy of the FE predictors. For the most optimised protocol, logistic regression showed aBMD_Neck_, MPhyS and MPatS to be significantly associated with the facture status, with AUC of 0.75, 0.75 and 0.79, respectively.

**Conclusion:**

The study emphasized the necessity of modelling the whole femur anatomy to develop a robust FE-based tool for hip fracture risk assessment. FE-strength performed only slightly better than the aBMD in discriminating the fracture and control cases. Differences between the published studies can be explained in terms of differences in the modelling protocol and cohort design.

## Introduction

Osteoporosis is characterized by a reduction in bone mineral density (BMD), which leads to an increased risk of fracture [1, 2]. The current clinical standard to estimate the risk of hip fracture is based on areal BMD (aBMD) measured by dual-energy absorptiometry (DXA), possibly combined with clinical risk factors in assessment tools like FRAX [3]. However, the predictive accuracy of aBMD is somehow limited and quite a few patients have experienced fractures in spite of being considered at low risk by this predictor. Quantitative computed tomography (CT)-based finite element (FE) models have been shown to predict the femoral strength as measured ex vivo with excellent accuracy [4–7]. But, when this CT-based, FE-estimated bone strength (hereinafter simply referred as FE-strength) is used to predict the risk of hip fracture, the few studies conducted have so far yielded contradictory results. While some studies concluded that FE-based strength was a much better predictor than aBMD [8, 9], in other studies, the improvement was much smaller [10, 11]. It is possible that some of these differences are due to the different modelling protocols used in the studies.

The aim of this work is to investigate if and how the FE modelling pipeline adopted to generate the subject-specific models affects the ability of the FE-strength estimated with such models in discriminating the fracture and the control groups in a retrospective cohort.

The FE modelling protocol developed in the European project VPHOP, which showed femur FE-strength to be a considerably better predictor of hip fracture than aBMD in a cohort of elderly women [6, 9], was adapted for the current study. The VPHOP protocol requires full-femur CT scans, constant CT parameters to allow off-line calibration and the use of a mesh morphing algorithm validated ex vivo to automate the modelling procedure [12].

The current study examined an age-, height- and weight-matched retrospective cohort of 100 postmenopausal women, half with a contralateral hip fracture and half without fracture. The FE-strength was used to discriminate between fracture cases and the controls. The data available in this retrospective cohort provided CT scans that covered only just below the lesser trochanter and were performed with variable current protocol; also, preliminary data suggested that the mesh morphing approach might not perform optimally for this study. First, we processed all cases in the cohort using a modelling pipeline that was as close as possible to that developed in the VPHOP project: we used proximal femur landmarks to define the reference system, performed an off-line calibration on the same CT system using average current values and generated the meshes using the mesh morphing procedure. Then, we revised the protocol introducing a statistical shape model to estimate the whole femur anatomy and defined the reference system using the full femur and replaced the mesh morphing with a standard individual automatic meshing algorithm. The effect of the two FE modelling protocols was compared in terms of discriminatory power of the FE-strength predictors that they provided. The results obtained on this cohort design were also compared with those reported by similar published studies, and the role of age matching was explored to understand the disparity of results among these published studies.

## Materials and methods

### Clinical cohort

The retrospective study was conducted on a cohort of 100 Caucasian women who were at least 5 years post-menopause. Fifty of these women had a hip fracture associated with low-energy trauma; the other 50, with no fracture, were selected to be pair-matched for age, height and weight. The inclusion criteria were as follows:

1. For fracture patients with the body mass index (BMI) between 16 and 34, the control was chosen to have age ± 5 years, height ± 5 cm and weight ± 5 kg.
2. For fracture patients with BMI ≥34 or BMI ≤16, the control was chosen to have age ± 5 years and BMI ± 4 kg/m^2^.

Sheffield Local Research Ethics Committee approved the study, and all subjects enrolled gave informed written consent. Details of this cohort are reported by Yang et al [13].

### Imaging protocol

All patients received a DXA scan (Hologic Discovery scanner, Hologic Inc, Bedford, MA, USA) according to the standard clinical protocol. aBMD values in the femoral neck (aBMD_Neck_) and total femur (aBMD_Total_) were derived for each patient.

All patients received a bilateral proximal femur CT scan (LightSpeed 64 VCT, GE Medical Systems, Milwaukee, WI, USA). The scanned region extended just above the femoral head to 3.5 cm below the lesser trochanter. All scans were performed at 120 kVp, with the tube current modulation (80–200 mA) and a slice thickness of 0.625 mm and pixel size of 0.74 × 0.74 mm^2^. Consistent with the protocol recommended by the VPHOP project, the European spine phantom (ESP) was scanned in the same CT scanner at 120 kVp/150 mA to derive the density calibration equations. Off-line calibrations can be affected by the stability of the X-ray tube. All patients were also scanned with an in-line K_2_HPO_4_ calibration phantom; across the 5 years that the clinical study took place, we found the differences between the in-line K_2_HPO_4_ calibration and the off-line ESP calibration to be less than 0.5 %, suggesting excellent stability of the tube.

The patient scans were performed using the automatic current modulation for dose reduction protocol [14], which, in principle, makes the off-line phantom calibration less accurate. However, a preliminary sensitivity analysis performed by scanning the ESP at three tube-current levels (100, 150 and 200 mA) showed differences in the femur strength never exceeding 3 %.

### FE modelling

#### Mesh generation

One femur (contralateral in fractured cases and matched controls) in each patient was segmented (ITK-Snap 2.0.0, University of Pennsylvania) to extract the three-dimensional bone morphology. The segmented bone surface was meshed with ten-node tetrahedral elements by employing two methods: a mesh morphing procedure [12] based on the anatomical landmarks on the femoral surface and a standard automatic meshing algorithm (ICEM CFD 14.0, Ansys Inc., PA, USA). The average element size was set to 3 mm following a convergence study [15].

Elastic moduli were mapped onto the meshed bone model (Bonemat, V3) using the relationships between radiological density (*ρ*_QCT_), ash density (*ρ*_ash_ = 0.877*ρ*_QCT_ + 0.079), wet apparent density (*ρ*_app_ = 0.6 *ρ*_ash_) and elastic modulus (*E* = 6.950*ρ*_app_^1.49^) [9, 16–18].

#### Femur reference system

A femoral reference system was generated to simulate different physiological loading scenarios. The reference system was defined by employing anatomical landmarks in the proximal femur scan only and was then updated using landmarks on an estimated full femur.

Full-femur anatomy was estimated from the segmented proximal femur using a rigid-body registration and a statistical shape model-guided fit. The reconstruction process was performed using the MAP Client software and associated plugins [19–21]. The proximal region of an averaged femur mesh was registered using the iterative closest point algorithm [22] to the segmented data cloud via a rigid-body transformation. The transformation was then applied to the averaged femur mesh to bring it into alignment with the data cloud. The femur mesh was then deformed along the principal components of a whole-femur shape model to minimise the least squares distance between each data point and its closest point on the mesh. The shape model ensured that the fitted whole femur accurately represented the proximal femur geometry and had a realistic shape overall.

**Fig. 1:**
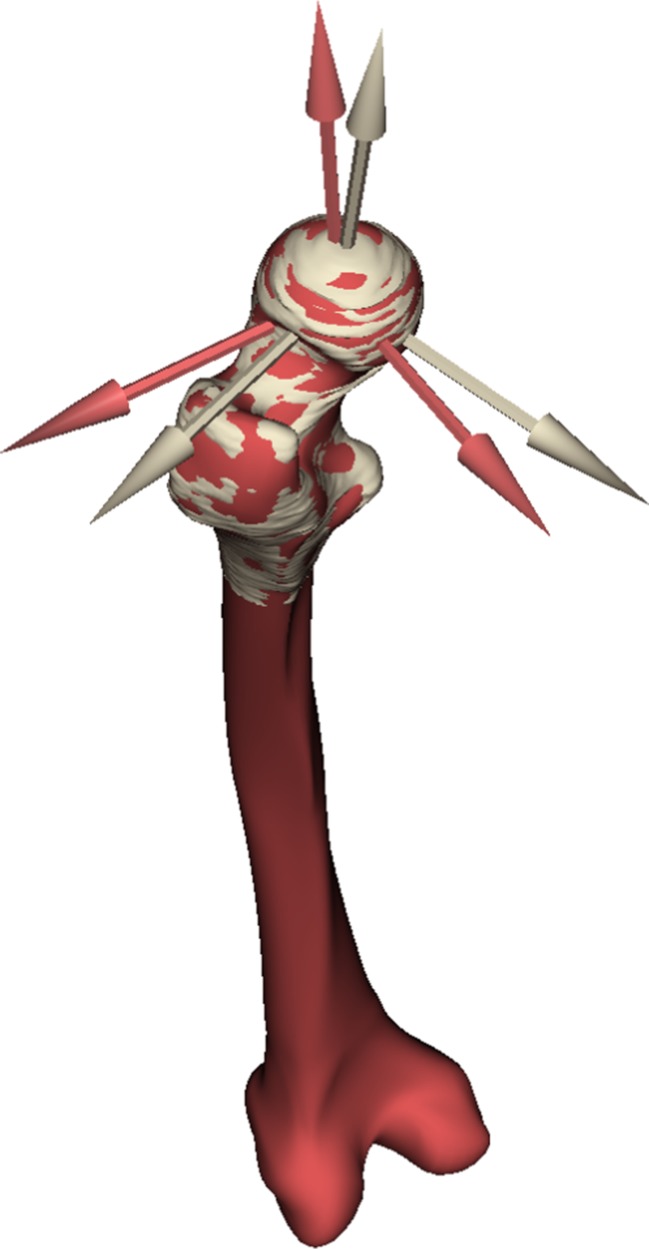
Variation in femoral reference systems representing the neutral stance position. Reference system in *brown colour* is generated by employing anatomical landmarks in the proximal femur while the one in *red colour* is based on the anatomical landmarks in the estimated full femur (Color figure online)

The femur orientation in neutral stance position was achieved by (i) defining a plane that passed through the centres of the femoral head, the neck and the diaphysis in the proximal femur and (ii) defining a plane tangential to the condyles, passing through the centre of the femoral head (Fig. 1).

#### Model variations

In total, four FE models were developed for each patient to investigate the role of meshing technique and femur orientation method in determining hip fracture risk: *model I*: morphed mesh/proximal femur orientation, *model II*: standard mesh/proximal femur orientation, *model III*: morphed mesh/full-femur orientation and *model IV*: standard mesh/full-femur orientation.

### Boundary conditions

#### Stance loading

**Fig. 2:**
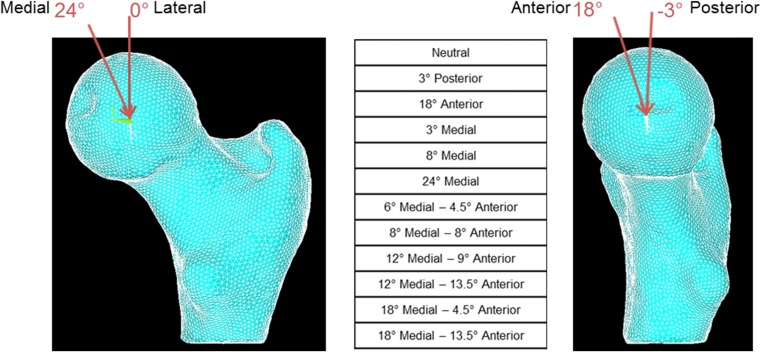
Variation of load direction in the frontal and sagittal planes representing a sample of daily activities. Twelve different loading conditions were simulated

Twelve different stance loading scenarios were simulated by varying the force direction from 0° to 24° in the frontal plane and −3° to 18° in the sagittal plane to account for different daily activities (Fig. 2) [9, 23].

#### Fall loading

**Fig. 3:**
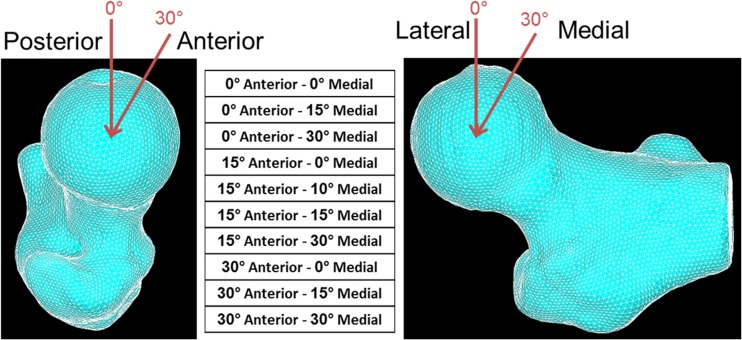
Variation of load direction in the frontal and transverse planes representing a sample of side fall loading scenarios. Ten different loading conditions were simulated

Ten different sideway fall loading scenarios were simulated by varying the force direction from 0° to 30° in the both frontal and transverse planes (Fig. 3) [9]. The fall loading analyses were performed using the modelling pipeline for model IV only, as it showed the best results for the stance loading analyses.

#### Constraints

A concentrated point load equal to patient’s body weight was applied at the centre of the femoral head, and the distal end of the femur was fully constrained in the both stance and fall loading scenarios. For fall loading scenarios, a no-friction slider was simulated by constraining the most lateral node on the greater trochanter in the loading direction while allowing it to translate in the other two directions.

### Femoral strength assessment

For each loading condition, the FE-strength was predicted for femoral neck fracture following a maximum principal strain criterion that has been validated in vitro and successfully applied in vivo [6, 9, 18, 24]. In short, FE-strength is the load that caused a principal strain greater than the limit value (0.73 % tensile limit strain, 1.04 % compressive) in the femoral neck surface [25]. Minimum physiological strength (MPhyS) was defined as the minimal FE-strength of the 12 stance loading conditions, and minimum pathological strength (MPatS) was defined as the minimal FE-strength of the ten fall loading conditions simulated. All FE analyses were performed using ANSYS14.0 (Ansys Inc, PA, USA).

### Statistical analyses

Mann-Whitney test (*α* = 0.05) was performed on patients’ weight, height, age, aBMD_Neck_, aBMD_Total_, T-scores, MPhyS and MPatS to determine the differences between the case and the control groups. Univariate logistic regression models were used to determine the ability of MPhyS, MPatS, aBMD, T-score and FRAX score to classify the control and cases. Odds ratio (OR) was calculated for a one standard deviation increase or decrease in the independent variable. The Hosemer-Lemeshow (H-L) test was used to test the goodness of fit for the models. Area under the receiver operating characteristic (ROC) curve (AUC) was calculated to compare the classifying power of the predictors. aBMD-adjusted regression models were employed to investigate the independent discriminatory power of the MPhyS and MPatS. Spearman correlations were calculated between the aBMD_Neck_, aBMD_Total_, MPhyS and MPatS. Statistical analyses were performed with SPSS21 (SPSS Inc., IBM, Chicago, IL, USA).

## Results

One case-control pair was excluded from the study because multiple high-density calcified areas were observed in the CT images of the fractured case. The results are thus reported for 98 individuals.

As each control was pair-matched to a fractured case, no significant differences were observed for patient’s age, weight and height between the two groups (Table 1). aBMD_Neck_ and aBMD_Total_ were significantly lower (15 %) for the fractured cases as compared to the control group (Table 2). Average T-scores and FRAX scores were also found to be significantly different between the two groups. The difference between the mean MPhyS of control and case groups was lowest for model I (8 %) and highest for model IV (22 %). MPatS was estimated for model IV only and was found to be, on average, 26 % higher for the control group than for the fracture group (Table 2).

**Table 1:** Patient’s characteristics classified into the control and case groups

	Controls (*N* = 49)	Cases (*N* = 49)	*p* value
	Mean (SD)	Mean (SD)	
Age (years)	75 (8)	75 (9)	0.592
Weight (kg)	64 (12)	62 (14)	0.712
Height (cm)	158 (5)	158 (7)	0.643

**Table 2:** Bone mineral density (BMD) and FE-strength estimates for control and case groups

		Controls (*N* = 49)	Cases (*N* = 49)	% Difference	*p* value
		Mean (SD)	Mean (SD)		
aBMD_Neck_ (g/cm^2^)		0.66 (0.10)	0.56 (0.10)	15	<0.0001
aBMD_Total_ (g/cm^2^)		0.80 (0.10)	0.68 (0.10)	15	<0.0001
T-score		−1.0 (1.0)	−2.1 (1.3)	–	<0.0001
FRAX score		9.1 (8.3)	14.7 (10.8)	–	0.001
MPhyS (N)	Model I	4139 (1212)	3823 (1321)	8	0.173
	Model II	4208 (1136)	3722 (1265)	11	0.01
	Model III	4374 (1001)	3489 (1022)	20	<0.0001
	Model IV	4446 (951)	3451 (981)	22	<0.0001
MPatS (N)		2729 (521)	2027 (698)	26	<0.0001

Logistic regression showed aBMDs, T-score, FRAX score and MPhyS/MPatS to be significantly associated with the fracture status (Table 3). AUCs for aBMD_Total_ and aBMD_Neck_ were 0.74 and 0.75, respectively. AUC for MPhyS increased from 0.59 to 0.65, 0.72and 0.75 for models I, II, III and IV, respectively. AUC for MPatS (0.79) was the highest among all the predictors while FRAX had the lowest AUC of 0.69. OR of 4.5 (2.3–9.0) for MPatS was also considerably higher than the other predictors (Table 3). aBMD-adjusted regression analyses showed MPhyS (*p* = 0.005) and MPatS (*p* = 0.002) to remain significantly associated with the fracture status (Table 3). MPhyS was not found to be correlated with either aBMD_Neck_ (*r* = 0.49) or aBMD_Total_ (*r* = 0.41), MPatS was found to be slightly correlated with aBMD_Neck_ (*r* = 0.69) and aBMD_Total_ (*r* = 0.62), while the two aBMDs were highly correlated (*r* = 0.91). MPhyS and MPatS were also found to be only slightly correlated with each other (*r* = 0.67).

**Table 3:** Logistic regression, odds ratios and area under the ROC curve for BMDs and MPS from model IV

	Odds ratio (OR)	95 % Confidence interval (CI)	AUC	*p* value
aBMD_Neck_	3.1	1.8–5.3	0.75	<0.0001
aBMD_Total_	2.8	1.7–4.6	0.74	<0.0001
T-score	2.6	1.6–4.3	0.74	<0.0001
FRAX score	1.9	1.1–3.2	0.69	0.017
MPhyS	3.2	1.8–5.6	0.75	<0.0001
MPatS	4.5	2.3–9.0	0.79	<0.0001
MPhyS BMD adjusted	2.3	1.3–4.2	0.79	0.005
MPatS BMD adjusted	3.2	1.4–7.3	0.80	0.002

## Discussion

The aim of this work was to investigate if and how the FE modelling pipeline adopted to generate the subject-specific models affected the ability of the FE-strength estimates obtained with such models in discriminating the fracture and control groups in a retrospective cohort.

The results support the development of FE models for hip fracture risk assessment while calling attention to the imaging protocols and the meshing techniques employed. The results showed the necessity of the whole femur anatomy in estimating the femur strength correctly. The pathological strength performed better than the physiological strength in discriminating the fracture and the control groups, which is supported by the fact that majority of the hip fractures are associated with a fall. The FE-strength performed only slightly better than the aBMD, with no considerable improvements as reported in the previous study [9]. This could be due to the sub-optimal imaging protocol employed in the retrospective cohort.

The mesh morphing algorithm employed in this work was reported to predict strains as measured on human cadaver femurs with excellent accuracy [12]. However, when applied in the current study, the morphed meshing technique reduced the power of FE-strength to discriminate between the control and the fracture cases. While the potential usefulness of technologies that automate the transformation of CT data into FE models is essential for the future wider adoption of these techniques, these results suggest caution in adopting new technologies, which should always be tested with regard to their discriminatory power over retrospective cohorts.

It is important to orient the femur in the physiological position to perform multiple loading analyses. As each patient’s position in the CT scanner is slightly different, anatomical landmarks were used to define the orientation of the femur. In current clinical practice, the CT scan of the hip is frequently limited to the proximal femur region. However, the proximal femur lacks some important anatomical landmarks, particularly those necessary to define the anteversion of the femoral neck. A considerable increase in the predictive power of FE-strength was observed by estimating the physiological reference system of the femur using a statistical shape model. Euler angles between the proximal and full-femur reference systems were found to vary among the patients (*θ*_*x*_ = 12° ± 7°, *θ*_*y*_ = 7° ± 3°, *θ*_*z*_ = 3° ± 3°), thus making it difficult to define a transformation function between the two reference systems. When retrospective cohorts with proximal-only CT have to be analysed, the proposed method can be useful; still, for prospective studies, full-femur CT should be preferred.

We recommend developing CT scan protocols that include the whole femur and extend to below the knee in order to obtain detailed anatomical information (in the distal femur) for anatomical landmarking. One of the concerns for such a protocol is the exposure of the patient to additional radiation dose. We used the ImPACT CT Patient Dosimetry Calculator to compare the effective radiation dose for a proximal and a whole femur scan. It makes use of the “Normalised organ doses for X-ray computed tomography calculated using Monte Carlo techniques” NRPB-SR250 released by Public Health England on Nov. 2014 and based on the organ weighting scheme ICRP-103. Effective radiation dose for the current clinical protocol for proximal femurs scan is in the range of 2.5–6.2 mSv for males and 1.5–3.8 mSv for females. By reducing the tube voltage from 120 to 100 kV, while keeping the remaining scan settings the same, the whole femur scan would result in an effective radiation dose in the range of 1.9–4.8 mSv for males and 1.3–3.2 mSv for females. While further work (e.g. using cadaveric bones) is required to ensure that reducing the tube voltage would not compromise the quality of the CT image and, consequently, the FE modelling, preliminary results based on phantoms show negligible differences (less than 0.1 % in density estimation) when this small voltage reduction is adopted.

Of the few similar studies reported in the literature, the most relevant to compare is that conducted using the same patient-specific modelling technologies developed in the European project VPHOP [9]. An important difference between that and the present study is that the data were collected following the recommendations of the VPHOP project (full-femur CT scan, constant energy scan); also, it employed standard automatic mesh generation instead of the mesh morphing technology. In this study, we quantified the effect of mesh morphing vs. standard meshing, and we used a statistical femoral atlas to minimise the effects of limited scan range (proximal femur only); we also confirmed that tube current variability had an effect of 3 % or less. However, even after we reduced as many of the limitations imposed by the data as possible in this study, our predictive accuracy is significantly lower than that presented by Falcinelli et al. who reported a substantially higher predictive power for MPhyS (AUC = 0.87) and MPatS (AUC = 0.88) for a cohort of 55 patients (cases 22, controls 33).

The difference between the mean MPatS for control and fracture groups was 29 % for their cohort as compared to 26 % in the current cohort. One reason for the difference between the two cohorts could be that current cohort was age-matched, while the controls in their cohort were, on average, 11 years younger than the cases. Similarly, Orwoll et al [8] reported a difference of 36 % in the mean FE femoral strength between case and control groups in a cohort of 250 men (cases 40, controls 210) with a difference in age of 5.5 years on average. While for an age-, height- and weight-matched cohorts of 139 males (cases 45, controls 94) and 170 females (cases 71, controls 145), differences in the mean stance failure load of control and case groups were reported to be 16 and 20 %, respectively [10]. For the current cohort, we explored the effect of age matching by removing any cases younger than 65 years and controls older than 80 years, effectively breaking the age-matched case and control groups. This resulted in a cohort of 71 patients (cases 39, control 32) with a difference in mean age of 10 years. The difference in the mean MPatS of control and cases increased to 29 % resulting in an AUC of 0.84. Age has been shown to highly correlate with the fracture risk in other clinical studies [26, 27]. In addition, the use of whole femur CT scans in the FE modelling and the use of constant current protocol which improves the off-line densitometric calibration could also contribute to the better classification reported by Falcinelli et al [9]. Also, small sample sizes (less than 100) have been shown to have more pronounced effect estimates than with larger samples [28, 29].

The cohort of this study was designed to reflect the typical distribution of osteopenia found in the population referred to an osteoporosis specialist in a secondary care setting; in various national guidelines, patients with a T-score >−1.5 are automatically assigned to the no-treat arm and referred for a further visit 2 years later. A cohort with a different distribution would have resulted in similar conclusions. The risk of fracture is due to three determinants: the total mineralised mass (measured by the BMD), the anatomical organisation of such mass in relation to the loading (measured by the FE-strength) and the loading itself, associated to the propensity to fall and overload. For patients with a BMD higher than that in our cohort, who are at risk for the anatomical determinant, it is easy to speculate that MPhyS and MPatS will perform much better than BMD as a predictor; on the contrary for patients with a BMD higher than that in our cohort, normal anatomy, but are at high risk because of their propensity to fall, we would expect neither the BMD nor MPhyS/MPatS to be accurate predictors.

Our results suggest that CT-based patient-specific FE models are more accurate than aBMD measurements in predicting the strength of a patient’s femur. BMD-adjusted logistic regression confirms that FE-strength contains additional and unique information in comparison to that captured by the aBMD. However, the risk of fracture depends on the bone strength as well as the incidence of overloading (i.e. falling) while the current analysis only captures the bone strength.

In summary, the current study emphasizes the importance of imaging protocol and FE modelling procedure for developing a robust and reliable diagnostic tool to predict osteoporotic hip fracture risk. It also suggests that CT-based FE-estimated strength is a mature and reliable predictor, which, when computed with optimal protocols, performs better than the aBMD in discriminating fracture and control cases.
